# The Mechanism of Motion

**Published:** 1896-01-25

**Authors:** 


### THE MECHANISM OF MOTION.

IN pictures of battles or of horse-races that noblest of animals is always represented with streaming mane, outstretched limbs, and body nearly touching the ground. This is our impression of a galloping horse —perhaps it is a fair likeness of its superficial aspect, but more probably it represents rather an ideal of strain and intensity which dwells in our own imagination. The photographic camera, that prime disillutionist, has proved that the real movements of the horse are not at all like those popularly represented, and it is doubtful if a true photograph of a horse galloping would not give an impression of slowness rather than speed. When thus analysed the component parts of any completed movement seem very unlike our idea of the movement itself. Yet that they are really its constituents may be easily shown if we can, so to speak, join them together again in the sequence in which they were dissected, as is done in the toy called the zoetrope.

The toy of the last generation has fallen into the hands of scientists, and by the aid of photography the rough representation of motion has been developed into the most exquisite analysis of direction, attitude, time, and space. Successive photographs taken at intervals of a fraction of a second present the different phases of a man's walk, a horse's trot, or a bird's flight, with a detail which is far beyond the power of the human eye, The wonders of this kind of photography are set forth in an interesting volume by M. E. J. Marey, which forms a sort of fairy-tale for biologists and artists.* It is needless to say that it deals with the most delicate experiments executed by instruments which are masterpieces of ingenuity. To follow the curves of a bird's wing in flight, a bright spangle is fastened to one of the longest quills or *remiges*. The bird is then allowed to fly towards a dark background, and the camera is brought to bear upon it. The bird and the background being dark, all the light which is thrown on the sensitised plate comes from the reflection of the brilliant spangle, which in its movements thus traces on the photograph the interlacing curves — not the straight up and down movement the eye imagines— taken by the wing as the bird flies onward. This continuous photograph gives a diagram of the bird's flight, but to show the actual attitudes the animal takes as it proceeds requires a more elaborate machine.

This is called the "photographic gun"—a weapon much like a large revolver in appearance, but charged with sensitised films instead of cartridges. The gun can be "fired," so to speak, at intervals of one-twelfth of a second, and each shot means the exposure for an instant of the recording film, after which the shutter falls again, to rise with a new film behind it, ready to picture the next development of motion. The photographs thus taken can be thrown on a screen by means of the limelight if public demonstration is desired, and if the films are placed above each other in swift enough succession, the spectator can see the whole movement reconstructed before his eyes. This method of chronophotography, as it is called, has been used in analysing the movements of the human body.

The first student of animal locomotion was Borelli, a Neapolitan professor of the seventeenth century, who applied to it the mechanical laws discovered by Galileo. He showed that the effect of muscular force made itself felt partly on the mass of the body, and partly on the point of resistance. This is, in its simplest term, the theory of locomotion. But, to obtain any degree of accuracy, it is necessary to take into account also the range, velocity, and sequence of any movement. Two brothers of the name of Weber, celebrated mathematicians in their day, carried on Borelli's experiments. But, limited as they were by inefficient measurements, their inferences were sometimes wrong. For instance, they enunciated, as the result of their studies, that "the length of the stride became greater as the frequency increased." This is true to a certain extent, but M. Marey's experiments, conducted by the aid of his better instruments, show that while a "quick march" means a greater stride as well as a quicker sequence of step than a slow processional march, the rate of progression diminishes when the steps exceed 85 per minute. Another observer, M. Demery, fancies he can test fatigue by means of pneumatic signals. In ordinary walking one foot leaves the ground as the other touches it, but there is a space, however short, when both feet are on the ground simultaneously. This space becomes greater as the pedestrian becomes fatigued, and finds an increasing difficulty in dragging one foot after the other.

But what, when all is said, is the use of this study? This, that by means of these accurate registrations and photographs of movements, or rather of the components of movements, we may learn how to attain the results obtained by those who have been thus studied and pictured. The athlete, the artist, the skilled artisan, can very rarely explain the motion of the body by which he does his work and produces the effect he desires. If he attempts it, his explanation is as often as not incorrect. Teachers of gymnastics tell their pupils, when they come to the ground after a jump, to land on their toes, in order to break the shock. Chrono-photographs taken by M. Marey show, on the contrary, that an athlete really lands on his heels after a jump; and this is only one example of misleading instruction concerning an action that has not been analysed. Doubtless, in many more serious industries we should find that the student or apprentice learns not by what his master tells him, but by watching results, and working out as best he can the means by which these are attained. How much simpler it would be if there were photographs of every part of a stroke to demonstrate clearly how it should be done. So far, the results of chrono-photography are experimentaland tentative; but even the amusements and the toys of the wise are helpful to the world, and these studies of motion, while they arouse the interest and satisfy the curiosity of the philosopher, may yet come— as such things have done before—to aid in the development and multiply the conveniences of the practical man.

* "Movement." By E. J. Marey, member of the Institute and of the Academy of Music; Professor at the College of France, Director of the Physiological Station. Translated by Eric Pritchard, M.A., M.B., B.Ch. Oxon. (London: Wm. Heinemann. 1895.)

